# Enhanced Polar Lights Optimization with Cryptobiosis and Differential Evolution for Global Optimization and Feature Selection

**DOI:** 10.3390/biomimetics10010053

**Published:** 2025-01-14

**Authors:** Yang Gao, Liang Cheng

**Affiliations:** School of Petroleum Engineering, Yangtze University, Wuhan 430100, China; gyang66_ytu@163.com

**Keywords:** polar lights optimization, global optimization, feature selection, differential evolution, cryptobiosis mechanism, bionic algorithm

## Abstract

Optimization algorithms play a crucial role in solving complex problems across various fields, including global optimization and feature selection (FS). This paper presents the enhanced polar lights optimization with cryptobiosis and differential evolution (CPLODE), a novel improvement upon the original polar lights optimization (PLO) algorithm. CPLODE integrates a cryptobiosis mechanism and differential evolution (DE) operators to enhance PLO’s search capabilities. The original PLO’s particle collision strategy is replaced with DE’s mutation and crossover operators, enabling a more effective global exploration and using a dynamic crossover rate to improve convergence. Furthermore, a cryptobiosis mechanism records and reuses historically successful solutions, thereby improving the greedy selection process. The experimental results on 29 CEC 2017 benchmark functions demonstrate CPLODE’s superior performance compared to eight classical optimization algorithms, with higher average ranks and faster convergence. Moreover, CPLODE achieved competitive results in feature selection on ten real-world datasets, outperforming several well-known binary metaheuristic algorithms in classification accuracy and feature reduction. These results highlight CPLODE’s effectiveness for both global optimization and feature selection.

## 1. Introduction

The increasing importance of feature selection arises from the complexities introduced by high-dimensional datasets [1]. In such datasets, irrelevant or redundant features can obscure meaningful patterns, compromise model performance, and escalate computational demands [2]. By concentrating on the identification of a subset of features that maintains or enhances a model’s predictive power, feature selection boosts the efficiency and efficacy of machine learning workflows [3].

Feature selection methods can be broadly categorized into three main types: filter methods, embedded methods, and wrapper methods, each distinguished by their underlying principles and inherent trade-offs [4]. Filter methods employ statistical measures to assess and rank features independent of any specific predictive model. Widely used techniques encompass correlation coefficients [5], mutual information [6], and variance thresholds [7]. Although computationally efficient, filter methods often overlook xinteractions among features, limiting their effectiveness in more complex situations. Embedded methods, conversely, integrate the feature selection process directly within the model training phase. Examples include Lasso regression [8], which introduces a penalty term to shrink the coefficients of less relevant features to zero and where feature importance is derived from split criteria. These methods generally provide improved performance by aligning feature selection with the model’s objectives but are restricted by the choice of the base algorithm. Wrapper methods take a more comprehensive and iterative approach, evaluating feature subsets using a predictive model [9]. Despite their computational overheads, they are effective at addressing feature interactions and customizing the selected subset for a particular problem. Techniques like forward selection, backward elimination, and recursive feature elimination illustrate this category, underscoring its ability to effectively optimize feature sets. The very nature of wrapper-based feature selection represents a global optimization problem, where the search for the optimal subset within an exponentially growing number of combinations necessitates the use of efficient algorithms. Formally, this problem can be defined as follows:

Let $ℱ=\{f_{1},f_{2},\ldots ,f_{n}\}$ denote the complete set of n features, and let $x=(x_{1},x_{2},\ldots ,x_{n})$ be a binary vector where $x_{i}\in \{0,1\}$ indicates whether the i-th feature is selected ($x_{i}=1$) or not ($x_{i}=0$). The goal of wrapper-based feature selection is to identify the optimal subset of features 𝒮⊆ℱ that maximizes (or minimizes) a predefined objective function J(𝒮), which typically evaluates the performance of a predictive model trained on the selected features. The search space for this problem is combinatorial in nature, with a total of $2^{n}$ possible feature subsets.

Traditional methods, such as exhaustive search or greedy algorithms, often struggle with the curse of dimensionality, thereby prompting the adoption of metaheuristic approaches [10].

Metaheuristic algorithms have emerged as effective tools for addressing challenging optimization problems, particularly in high-dimensional, multimodal, and non-convex search spaces [11]. These algorithms can be generally categorized into two main types: evolutionary algorithms and swarm intelligence algorithms. Evolutionary algorithms, drawing inspiration from natural selection, encompass techniques such as genetic algorithms (GA) [12] and differential evolution (DE) [13], which emulate biological evolutionary processes. Conversely, swarm intelligence algorithms, inspired by the collective behaviors of animal groups, include methods like particle swarm optimization (PSO) [14] and ant colony optimization (ACO) [15]. While both categories emphasize the importance of balancing exploration and exploitation, they diverge in their foundational principles and operational mechanisms.

Over the past few years, metaheuristic algorithms, particularly swarm intelligence-based approaches, have shown significant promise in addressing FS challenges. Several studies have explored improved versions of established metaheuristic algorithms for FS. For instance, Gao et al. [16] introduced clustering probabilistic particle swarm optimization (CPPSO), which enhances traditional PSO with probabilistic velocity representation and a K-means clustering strategy to improve both exploration and exploitation for high-dimensional data. Similarly, hybrid approaches have gained traction, such as the particle swarm-guided bald eagle search (PS-BES) by Kwakye et al. [17], which combines the speed of PSO to guide bald eagle search, introducing an attack–retreat–surrender mechanism to better balance diversification and intensification. These studies showcase the effectiveness of leveraging different search mechanisms for improved performance on benchmark datasets and real-world problems. Other variations of metaheuristics have explored improved exploration strategies, such as a modified version of the forensic-based investigation algorithm (DCFBI) proposed by Hu et al. [18], incorporating dynamic individual selection and crisscross mechanisms for improved convergence and avoidance of local optima. Furthermore, Askr et al. [19] proposed binary-enhanced golden jackal optimization (BEGJO), using copula entropy for dimensionality reduction while integrating enhancement strategies to improve exploration and exploitation capabilities. Beyond the improved variations of metaheuristic algorithms, novel algorithms have also emerged. Lian et al. [20] presented the parrot optimizer (PO), inspired by parrot behaviors, integrating stochasticity to enhance population diversity and avoid local optima. Likewise, Singh et al. [21] explored combining emperor penguin optimization, bacterial foraging optimization, and their hybrid to optimize feature selection for glaucoma classification. These studies indicate the emergence of diverse metaheuristic strategies to balance exploration and exploitation for FS.

While metaheuristic approaches have shown considerable success in feature selection, the no free lunch (NFL) theorem highlights their inherent limitations [22]. The NFL theorem asserts that no single optimization algorithm can consistently outperform all others across all problem instances. This necessitates ongoing innovation and adaptation of metaheuristic strategies to address diverse feature selection challenges. Researchers are thus motivated to refine existing algorithms or explore the combination of multiple techniques, such as hybridizing algorithms or incorporating adaptive mechanisms, to enhance their generalizability and robustness [23,24]. Informed by these considerations and the need to overcome the constraints of current metaheuristic algorithms, this study introduces an innovative approach to enhance existing algorithms, aiming to advance their applicability to both feature selection and global optimization tasks.

The polar lights optimization (PLO) algorithm, a recent metaheuristic optimization approach proposed by Yuan et al. in 2024 [25], draws its inspiration from the natural phenomenon of the aurora. PLO emulates the movement of high-energy particles as they are affected by the Earth’s magnetic field and atmosphere, incorporating three fundamental mechanisms: gyration motion for local exploitation, aurora oval walk for global exploration, and particle collision to facilitate an escape from local optima. A key advantage of PLO lies in its ability to balance local and global search through the use of adaptive weights. However, similar to other metaheuristic algorithms, PLO’s performance can be susceptible to parameter settings, and its convergence may be challenged by high-dimensional problems. Therefore, further research is warranted to investigate parameter-tuning strategies and assess PLO’s performance across diverse real-world applications to validate its robustness and practical utility.

This paper introduces CPLODE, an enhanced version of the polar lights optimization (PLO) algorithm, designed to improve its search capabilities through the integration of a cryptobiosis mechanism and differential evolution (DE) operators. Specifically, the cryptobiosis mechanism refines the greedy selection process within PLO, allowing the algorithm to retain and reuse historically effective search directions. Moreover, the original particle collision strategy in PLO is replaced by DE’s mutation and crossover operators, which provide a more effective means for global exploration and employ a dynamic crossover rate to promote improved convergence. These modifications collectively contribute to the enhanced performance of CPLODE. The key contributions of this paper can be summarized as follows:

- A novel enhanced polar lights optimization algorithm, CPLODE, is proposed by integrating a cryptobiosis mechanism and differential evolution operators to enhance the search effectiveness.
- The DE mutation and crossover operators replace the original particle collision strategy and use a dynamic and adaptive crossover rate to enable better solution convergence.
- The cryptobiosis mechanism replaces the greedy selection approach and allows for the preservation and reuse of historically successful solutions to improve the overall performance.
- The performance of CPLODE is validated through comprehensive experiments, demonstrating its efficacy in solving complex optimization problems.

The remainder of this paper is organized as follows: Section 1 introduces the research background, motivation, and key contributions. Section 2 describes the fundamentals of the original PLO algorithm. Section 3 presents the proposed CPLODE algorithm, including detailed explanations of the cryptobiosis mechanism and the DE operators. Section 4 covers the experimental setup, results, and their analysis to evaluate CPLODE’s performance. Section 5 explores the application of the proposed CPLODE algorithm in feature selection. Finally, Section 6 concludes the paper by summarizing the key findings and outlining potential future work.

## 2. The Original PLO

Polar lights optimization (PLO), introduced by Yuan et al. [25] in 2024, is a novel metaheuristic algorithm that mimics the movement of high-energy particles interacting with the Earth’s geomagnetic field and atmosphere, inspired by the natural phenomenon of the aurora. The algorithm solves optimization problems by modeling this particle motion, which is divided into three core phases: gyration motion, aurora oval walk, and particle collision.

**1. Gyration motion:** Inspired by the spiraling trajectory of high-energy particles under Lorentz force and atmospheric damping, gyration motion facilitates local exploitation. Mathematically, this is represented by the following equation:

(1) $$v\left(t\right)=Ce^{\frac{qB-a}{m}t}$$

where $v\left(t\right)$ represents the particle’s velocity at time t, C is a constant, q represents the particle’s charge, B is the strength of Earth’s magnetic field, α represents the atmospheric damping factor, and m is the mass of the particle. In the PLO algorithm, C, q, and B are set to 1 for simplicity, and *m* is set to 100. The damping factor α is a random value within the range [1, 1.5]. The fitness evaluation process of the current particle represents the time (t) to model the decaying spiraling trajectories, enabling fine-grained local searches.

**2. Aurora Oval Walk:** The aurora oval walk emulates the dynamic movement of energetic particles along the auroral oval, facilitating global exploration. This movement is influenced by a Levy flight distribution, the average population position, and a random search component. The aurora oval walk of each particle is calculated using the following equation:

(2) $$Ao=Levy(d)\times (X_{avg}\left(j\right)-X\left(i,j\right))+LB(i)+r_{1}\times (UB(i)-LB(i))/2$$

where Ao represents the movement of a particle in the auroral oval walk, i represents the i-th individual and ranges from 1 to N (population size), and *j* represents the j-th dimension, ranging from 1 to D (problem dimension). Levy(d) is the Levy distribution, d is the step size, $\left(X_{avg}(j)-X(i,j)\right)\;$ represents the direction the particles tend to move toward the average location, LB is the lower bound of the search space, UB is the upper bound of the search space, and $r_{1}$ is a random number [0, 1].

This auroral oval walk enables rapid exploration of the solution space through a seemingly random walk. To integrate both gyration motion and the auroral oval walk, the updated position of each particle ($X_{new}(i,j)$) is computed as follows:

(3) $$X_{new}(i,j)=X(i,j)+r_{2}\times (W_{1}\times v(t)+W_{2}\times Ao)$$

where X(i,j) is the current particle position, and $r_{2}$ introduces randomness, taking values between 0 and 1. $W_{1}$ and $W_{2}$ are adaptive weights that balance exploration and exploitation. They are updated in each iteration as follows:

(4) $$W_{1}=\frac{2}{\left(1+e^{-2(t/T{)}^{4}}\right)}-1$$

(5) $$W_{2}=e^{-(2t/T{)}^{3}}$$

where t is the current iteration, and T is the maximum number of iterations. $W_{1}$ increases over time, giving more weight to gyration motion and $W_{2}$ decreases, giving less weight to the auroral oval walk, shifting from global search towards local exploitation.

**3. Particle Collision:** Inspired by the violent particle collisions in Earth’s magnetic field, which result in energy transfer and changes in particle directions, this strategy enables particles to escape local optima. In PLO, each particle may collide with any other particle in the swarm and is modeled mathematically with:

(6) $$X_{new}\left(i,j\right)=\left\{\begin{array}{ll} X\left(i,j\right)+\sin\left(r_{3}\times \pi \right)\times \left(X\left(i,j\right)-X\left(a,j\right)\right) & r_{4}<K\;and\;r_{5}<0.05\\ X(i,j) & otherwise \end{array}\right.$$

where $X_{new}\left(i,j\right)$ is the new position of particle i in dimension j, $X\left(i,j\right)$ is the current position of particle i in dimension j, $X\left(a,j\right)$ is the position of a randomly selected particle in the population, and $r_{3}$, $r_{4}$, and $r_{5}$ are random numbers from [0, 1]. The sine function introduces a variable direction of movement after the collision. The collision probability, K, increases with iterations as follows:

(7) $$K=\sqrt{\left(t/T\right)}$$

**Figure 1:**
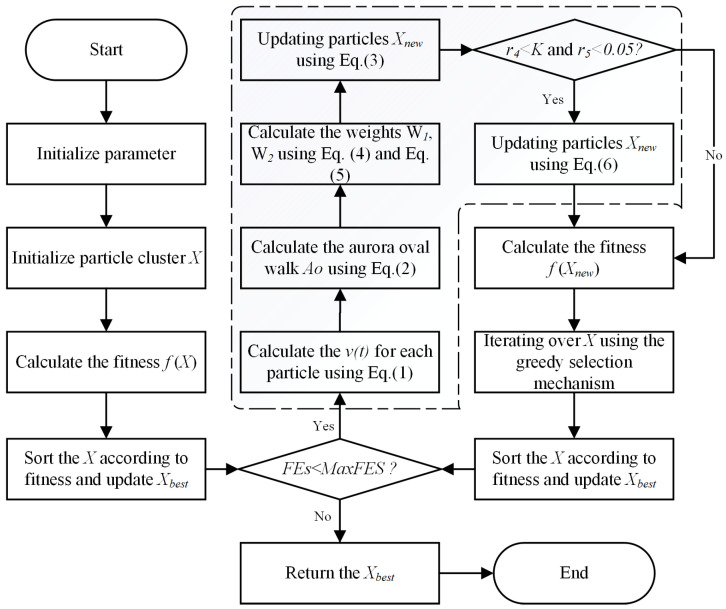
Flowchart of PLO.

The PLO algorithm iteratively updates the particle positions by combining gyration motion and the aurora oval walk by Equations (2) and (3). These motion patterns are balanced by adaptive weights that gradually shift emphasis from global exploration to local exploitation. The particle collision behavior occurs stochastically, allowing particles to escape local optima. This process continues until a maximum number of iterations is reached, resulting in a near-optimal solution. The core strength of PLO lies in the combination of these inspired physical behaviors to enable an effective search and a specific mechanism to avoid local optima. Figure 1 shows the flowchart of PLO.

## 3. Proposed CPLODE

### 3.1. Differential Evolution

Differential evolution (DE) is a population-based evolutionary algorithm known for its effectiveness in solving optimization problems through mutation and crossover operators [13]. In this work, we leverage DE’s mutation and crossover operators as an alternative to the particle collision strategy in the original PLO algorithm, aiming to enhance the algorithm’s global exploration capability. This replacement provides a more effective solution generation strategy than the random collisions used previously. Furthermore, the $r_{4}<K\;\mathrm{and}\;r_{5}<0.05$ condition for particle collision is replaced with a dynamic and adaptive crossover rate. The specific implementation of these DE operators within the improved PLO is detailed below.

1. Mutation: The mutation operator, crucial in DE, generates a trial vector by perturbing the current solution. We employ the “DE/best/1” mutation strategy [26], which perturbs a base vector by adding a scaled difference vector. This is mathematically expressed as:

(8) $$M(i)=X_{best}+F\times \left(X\left(r1\right)-X\left(r2\right)\right)$$

where $X\left(r1\right)$ and $X\left(r2\right)$ are two randomly selected individuals from the population, $X_{best}$ is the best individuals in the population, and F is a scaling factor.

2. Crossover: Following mutation, a crossover operator is used to increase population diversity by combining the beneficial features. We employed a binomial crossover, where each component of the newly generated offspring was selected from either the mutated vector or the current solution with a crossover probability, Cr. This is mathematically expressed as:

(9) $$C(i,j)=\left\{\begin{array}{ll} M(i,j) & rand<Cr\\ X(i,j) & otherwise \end{array}\right.$$

where C(i,j) represents the jth dimension of the ith offspring, rand is a random number between 0 and 1, and Cr is the crossover rate, as shown in Equation (10).

(10) $$Cr={0.5e}^{-2(FEs/MaxFEs{)}^{1/2}}+0.1$$

where FEs represents the current number of fitness evaluations, and MaxFEs is the maximum number of fitness evaluations. This dynamic crossover rate, Cr, starts at a higher value initially, promoting exploration, and gradually decreases as the algorithm iterates, transitioning the algorithm from diversification to intensification. This allows the algorithm to effectively search the entire solution space initially and then focus on exploiting promising regions, thus enhancing convergence.

### 3.2. Cryptobiosis Mechanism

The cryptobiosis mechanism, proposed by Zheng et al. in 2024 as part of the moss growth optimizer (MGO) algorithm [27], is implemented to refine the greedy selection mechanism. Drawing inspiration from the cryptobiosis phenomenon observed in moss, which allows them to endure periods of inactivity and subsequently revive under favorable conditions, this mechanism records historical information for each solution. In contrast to conventional methods that directly modify individuals, this mechanism stores the solutions generated in each iteration. Specifically, it maintains a record of a fixed number of past solutions and tracks the best-performing particle. When specific criteria are met, such as reaching the maximum number of records or the conclusion of a generation, the mechanism is triggered. The best historical solution among the recorded solutions is then employed to replace the current solution. This approach facilitates repeated exploration of promising areas, thereby ensuring the population’s global search capability. Concurrently, replacing individuals with the best historical solutions under these conditions enhances population quality. This mechanism remains active throughout the search process, aiming to improve search efficiency by reintroducing previously successful solutions rather than initiating the search from scratch at each step.

The pseudo-code of the cryptobiosis mechanism is shown in Algorithm 1. In Algorithm 1, several variables are used to manage the cryptobiosis mechanism. $X\left(i\right)$ represents the i-th solution within the population; $rec_{num}$ denotes the maximum number of records that can be kept before a cryptobiosis event occurs; record is a counter tracking the number of records currently stored; $X_{record}(i)$ stores the recorded solutions for the i-th individual; t represents the current iteration, and T is the maximum number of iterations allowed before the next cryptobiosis cycle. $X_{record}^{best}(i)$ stores the best solution from the recorded solutions for the i-th individual. The algorithm cycles until the maximum number of fitness evaluations (MaxFEs) are reached. Within each cycle of the algorithm, solutions are recorded, and a local best solution is found within the recorded solutions.
**Algorithm 1:** Pseudo-code of cryptobiosis mechanism1.**Input:** $X\left(i\right)$: *i*-th solution rec_num: maximum number of records2.**Output:** Updated $X\left(i\right)$3.record=04.**While** (FEs<MaxFEs)5.         
**If** record=0
6.                 
$X_{record}(i)=X(i)$7.                 
record=record+1
8.         
**End if**
9.         
**Update** the X                                    /* PLO */
10.       
**For** *i* = 1:N
11.                 
$X_{record}(i)=X(i)$
12.                 
record=record+1
13.                 
**If** $record>rec_{num}-1||t\geq T$
14.                         
$X_{record}(i)=X(i)$
15.                         
**For** e=1:record
16.                                 
**If** $Fitness\left(X_{record}(i)\right)<Fitness(X_{record}^{best}(i))$
17.                                         
$X_{record}^{best}(i)=X_{record}(i)$
18.                                 
**End if**
19.                         
**End for**
20.                         
$X(i)=X_{record}^{best}(i)$
21.                         
record=0
22.                 
**End if**
23.         
**End For**
24.         
FEs=FEs+N
25.**End while**26.**Return** *X*

### 3.3. The Proposed CPLODE

**Figure 2:**
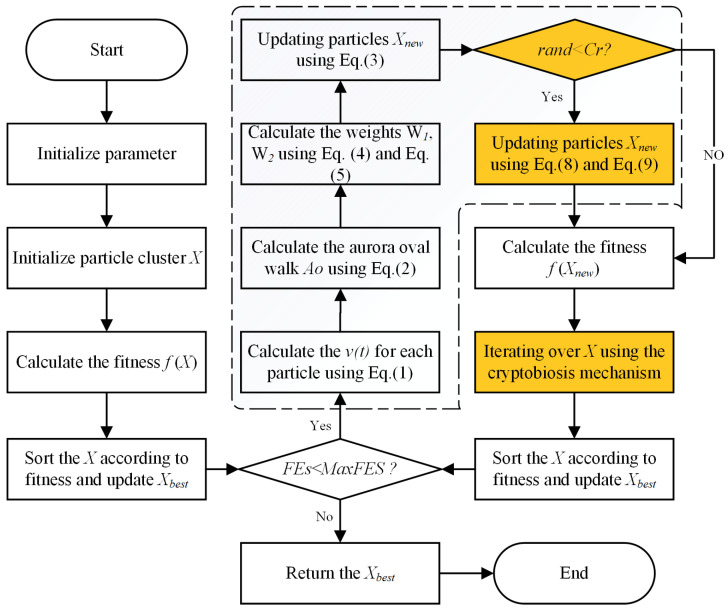
Flowchart of CPLODE.

This section delineates the workflow of the proposed CPLODE algorithm, which integrates the cryptobiosis mechanism and DE operators into the original PLO framework. CPLODE commences by initializing the required parameters and generating an initial population of solutions, which is consistent with standard optimization algorithms. The algorithm then proceeds through the following primary steps. Initially, the gyration motion strategy of PLO is executed to perform a local search around the current particle. Subsequently, instead of employing the original particle collision strategy, CPLODE leverages the DE mutation and crossover operators, as detailed in the preceding section, to produce new candidate solutions. Specifically, the “DE/best/1” mutation operator perturbs the current solution based on a scaled difference vector, while the binomial crossover operator, utilizing a dynamic crossover rate Cr, combines the mutated solution with the current solution. These steps ensure effective global exploration of the search space. Following the completion of gyration motion and mutation/crossover by all particles in the population, the cryptobiosis mechanism is activated. This mechanism records historical information for each particle throughout the previous iterations, and upon activation, it replaces the current solution with the best-recorded solution if a more effective historical solution is identified. The algorithm continues to iterate through these steps until a termination criterion is satisfied, at which point the algorithm returns the optimal or near-optimal solution. The overall workflow of CPLODE is illustrated in Figure 2.

Algorithm 2 provides the pseudo-code for the CPLODE.
**Algorithm 2:** Pseudo-code of CPLODEParameters initializing: FEs=0, MaxFEs, t=0
Initialize high-energy particle cluster X.Calculate the fitness value f(X).Sort X according to f(X).Update the current optimal solution $X_{best.}$**While** FEs<MaxFEsCalculate the velocity v(t) for each particle, according to Equation (1).Calculate aurora oval walk Ao for each particle, according to Equation (2).Calculate weights $W_{1}$ and $W_{2}$ according to Equations (4) and (5).**For** each energetic particle **do**Updating particles $X_{new}$ using Equation (3).**If** $r_{4}<K$ and $r_{5}<0.05$Particle collision strategy: update particle $X_{new}$ using Equations (8) and (9).**End If**          Calculate the fitness $f(X_{new})$.          FEs=FEs+1.**End For**  **If** $f\left(X_{new}\right)<f(X)$
          Iterating over X using the cryptobiosis mechanism.  **End If**Sort X according to f(X).Update the optimal solution $X_{best}$.                                                       t=t+1.**End While****Return the** $X_{best}$.

The computational complexity of the proposed CPLODE algorithm depends primarily on population initialization, fitness evaluation, gyration motion, DE-based solution generation, cryptobiosis mechanism, and the particle collision strategy. Assuming a population size of N, a maximum number of iterations of T, and a solution dimension of D, the overall computational complexity of CPLODE can be approximated as follows: O(CPLODE) ≈ O(TN)* + O(TND) + O(TND) + O(TN)* + O(TN)* ≈ O(TND). Therefore, the computational complexity of CPLODE is dominated by the DE-based solution generation and gyration motion and has a time complexity of O(TND).

## 4. Global Optimization Performance Evaluation

This section presents a comprehensive evaluation of the proposed CPLODE algorithm’s performance on a set of 29 benchmark functions from the IEEE CEC 2017 test suite. These experiments aim to provide a rigorous assessment of CPLODE’s optimization capabilities. All tests were conducted under standardized conditions on an Ubuntu 22.04 system using MATLAB R2023b, with a consistent configuration to ensure a fair comparison. The experiments were performed on an AMD Ryzen 9 5900X processor with 64 GB of RAM. To evaluate the algorithm’s performance, each algorithm was executed 30 times, and the average and standard deviation of the results for each benchmark function were recorded. For these experiments, the population size was set to 30, the problem dimension was set to 30, and the maximum number of fitness evaluations was set to 300,000. The following analysis will detail these results and provide an in-depth performance comparison.

### 4.1. Detailed Description of Benchmark Functions

The performance of the proposed CPLODE algorithm was evaluated using a suite of 29 benchmark functions from the 2017 IEEE Congress on Evolutionary Computation (CEC 2017) test suite [28]. These functions encompass a diverse range of characteristics, categorized into four primary types: unimodal, multimodal, hybrid, and composition functions. This selection of functions ensures a robust evaluation of the algorithm’s optimization capabilities across various landscape complexities. Each function is defined with a specific global optimum, as summarized in Table 1, which provides the function name, its type, and its optimal objective value. These benchmark functions serve as a standard tool to analyze and compare the effectiveness of optimization algorithms.

### 4.2. Comparative Analysis with Classical Optimization Algorithms

To evaluate the performance of the proposed CPLODE algorithm, comparative experiments were conducted against the following eight other classical optimization algorithms: PLO [25], SMA [29], WOA [30], GWO [31], MFO [32], SCA [33], FA [34], and DE [13]. These algorithms were chosen to provide a broad comparison across different optimization techniques. The experiments were performed on the 29 benchmark functions from the CEC 2017 test suite, and each algorithm was executed 30 times with a population size of 30, a solution dimension of 30, and a maximum of 300,000 function evaluations.

Table 2 summarizes the average (Avg) and standard deviation (Std) of the fitness values obtained by each algorithm on the 29 benchmark functions. Furthermore, Table 2 presents the overall rankings of each algorithm based on the Friedman test, along with the win/loss/tie results of CPLODE against each other algorithm. As shown in Table 2, CPLODE achieves the best average rank with a score of 1.6552, indicating its superior performance. In detail, CPLODE performs favorably on most functions and notably obtains results that are equal to or better than the other algorithms, especially on complex multimodal functions such as F3, F14, F17, and F19, demonstrating its effectiveness in navigating complex optimization landscapes.

Table 3 provides the *p*-values resulting from the Wilcoxon signed-rank test, which compares CPLODE against each of the other algorithms on the 29 benchmark functions. A *p*-value less than 0.05 indicates a statistically significant difference between the performance of CPLODE and the other algorithm. As shown in Table 3, the *p*-values for the majority of the functions are less than 0.05, demonstrating that CPLODE significantly outperforms the other algorithms. In those cases where the *p*-value is higher than 0.05, this typically indicates that CPLODE and that particular algorithm are able to provide results of comparable quality, typically when the problem is very simple, such as F12, F14, F21, F22, F23, F25, F26, F28, and F29.

The results presented in Table 2 and Table 3 demonstrate that CPLODE not only achieves a higher average rank but also exhibits a robust and consistent performance when compared to the selected classical optimization algorithms in these global optimization experiments.

**Figure 3:**
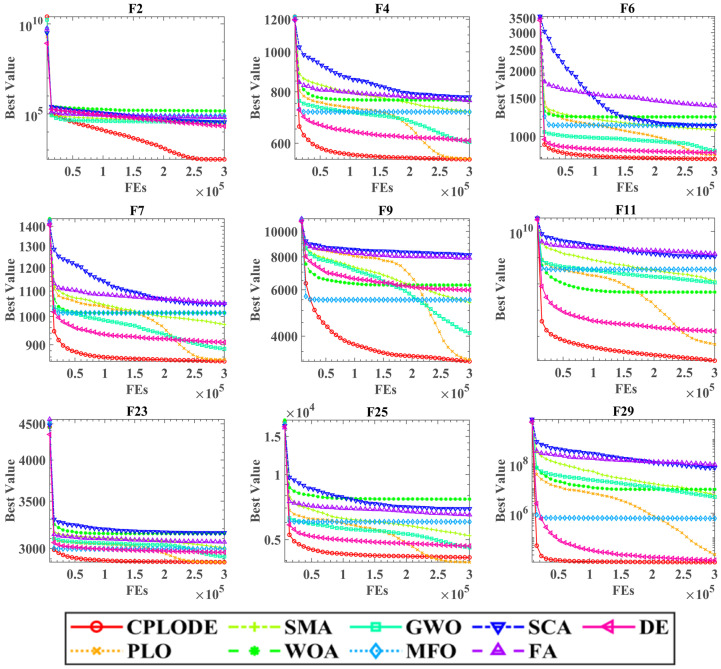
Convergence curves of CPLODE on benchmarks with other algorithms.

Figure 3 illustrates the convergence behavior of CPLODE and the other comparison algorithms across several representative benchmark functions (F2, F4, F6, F7, F9, F11, F23, F25, and F29). The horizontal axis represents the number of fitness evaluations (FEs) performed, while the vertical axis shows the best fitness value achieved by each algorithm at each evaluation. The legend at the bottom of the figure identifies each algorithm.

A visual analysis of the convergence curves reveals that CPLODE (the red line with circles) consistently achieves a superior convergence rate and reaches better fitness values compared to the other algorithms. Notably, for most of the shown functions, CPLODE exhibits a steep descent in fitness value during the initial evaluations, which indicates rapid convergence and demonstrates its strong exploitation capacity. The red lines consistently fall below the other colored lines across nearly all of the functions, showcasing that the CPLODE algorithm effectively navigates the search space and escapes local optima effectively to locate more promising solutions compared to all the comparison algorithms. Specifically on the more challenging functions, such as F7, F9, and F29, other algorithms are more prone to stagnating at local optima and show a much slower rate of convergence in comparison to the CPLODE. The performance demonstrates that CPLODE provides better exploration ability to navigate the entire solution space, and also has better exploitation capabilities, thus achieving faster and more reliable convergence.

## 5. Application to Feature Selection

This section explores the application of the proposed CPLODE algorithm to feature selection problems. Feature selection is a critical task in machine learning, aimed at identifying the most relevant features from a dataset, thereby reducing dimensionality and improving model performance. To apply CPLODE, a method designed for continuous domains, to this discrete problem, we employed a binary encoding strategy. Specifically, we constrained the problem’s upper and lower bounds to the interval [0, 1] and utilized Equation (11) to determine the selection status of each feature. This approach enables CPLODE, which is inherently suited for continuous domains, to operate effectively within the binary search space of feature selection.

(11) $$X_{i,j}=\left\{\begin{array}{c} 0\;X_{i,j}<0.5\\ 1\;X_{i,j}\geq 0.5 \end{array}\right.$$

To transition from the continuous search space of CPLODE to the binary feature selection space, each dimension is converted using a threshold of 0.5. Specifically, if a dimension’s value is greater than or equal to 0.5, the corresponding feature is selected; otherwise, it is not selected. This process maps the continuous space to a binary selection space.

In this study, we use the K-nearest neighbors (KNN) classifier to evaluate the quality of the selected feature subsets and use the following fitness function, which seeks to simultaneously minimize the classification error and the number of selected features:

(12) $$Fitness=\mu \;\times E+(1-\mu )\times \frac{l}{L}$$

where E represents the classification error rate, *l* is the number of selected features, *L* is the total number of features, and *μ* is a constant between 0 and 1 controlling the trade-off between the error rate and the number of selected features. Because we primarily focus on the accuracy of the selected feature subset, we set *μ* to 0.05 to prioritize error minimization. This setting gives more weight to the classification error and less weight to the number of features selected.

### 5.1. Detailed Description of Datasets

To evaluate the performance of the proposed CPLODE algorithm for feature selection, experiments were conducted on ten datasets selected from the UCI Machine Learning Repository. These datasets represent a range of complexity with varying numbers of samples, features, and classes. The number of samples in these datasets ranges from 72 to 2310, while the number of features varies from 8 to 7130. These variations ensure that the performance of the algorithm is tested across diverse feature selection scenarios, from low-dimensional to high-dimensional data and with different class distributions. Table 4 provides a detailed description of each dataset used in this study, including the dataset name, number of samples, number of features, and number of classes.

### 5.2. Feature Selection Results and Discussion

This subsection presents the experimental results of CPLODE for feature selection, compared with several other well-known binary metaheuristic algorithms as follows: BPSO [35], BGSA [36], BALO [37], BBA [38], and BSSA [39]. These experiments were conducted on the ten real-world datasets described in Section 5.1. All algorithms were run with a population size of 30 and a maximum of 1000 iterations. To ensure robustness and avoid bias, a 10-fold cross-validation technique was used in all experiments. The detailed experimental results are shown in Table 5 and Table 6.

Table 5 presents the average classification error rates, with standard deviations in parentheses, obtained by each algorithm on each dataset. From the table, it can be observed that CPLODE achieves the lowest error rates on the majority of datasets, demonstrating its superior performance in feature selection. Notably, CPLODE achieves significantly lower error rates on datasets such as “Hepatitis_full_data” and “Segment”, where the error rates of CPLODE are well below other binary metaheuristic algorithms. While some algorithms, such as BPSO and BSSA, perform well on datasets like “Leukemia”, their results are not consistently better than CPLODE across all datasets. On the “Heart” dataset, CPLODE, BGSA, and BSSA exhibited similar performances, but all of the algorithms outperformed BBA, which had the worst performance. These results indicate that CPLODE achieves better accuracy on many of the datasets presented.

Table 6 presents the average number of selected features, with standard deviations in parentheses, achieved by each algorithm on each dataset. From the table, it can be observed that while CPLODE shows a competitive ability in selecting the relevant features, it does not consistently select the shortest feature subsets. For example, the BGSA algorithm achieves lower feature selections on the “Hepatitis_full_data” and “Leukemia” datasets. However, this lower feature count comes at the expense of higher error rates, as shown in Table 5. Overall, CPLODE tends to select feature subsets that strike a good balance between classification performance and feature reduction, although not always selecting the absolute minimum number of features.

In summary, the experimental results demonstrate that the proposed CPLODE algorithm provides a highly competitive performance for feature selection. The proposed CPLODE demonstrates superior performance on most of the datasets used in this study, achieving a better classification performance. These superior results can be attributed to the integration of effective global search mechanisms through DE while employing the cryptobiosis mechanism for population quality control and the gyration motion strategy of PLO for local exploitation.

## 6. Conclusions

In this work, we have introduced CPLODE, a novel enhancement of the PLO algorithm, achieved through the integration of a cryptobiosis mechanism and DE operators. These modifications were designed to improve the original PLO’s search capabilities. Specifically, we replaced the original particle collision strategy with DE’s mutation and crossover operators, which enables more effective global exploration while also employing a dynamic crossover rate to enhance convergence. Furthermore, the cryptobiosis mechanism was incorporated to refine the greedy selection approach by recording and reusing historically successful solutions.

The performance of CPLODE was assessed on 29 benchmark functions from the CEC 2017 test suite, demonstrating superior performance across diverse fitness landscapes when compared to eight classical optimization algorithms. CPLODE achieved a higher average rank and statistically significant improvements, according to the Wilcoxon signed-rank test, particularly on more complex functions. Convergence curves further validated its enhanced convergence rate and optimal function values. These results emphasize the effectiveness of the integrated cryptobiosis mechanism and DE operators within CPLODE, confirming its improved search capability.

Moreover, CPLODE was applied to ten real-world datasets for feature selection, showcasing competitive performance by outperforming several well-known binary metaheuristic algorithms on most datasets and achieving a good balance between classification accuracy and feature reduction.

Future research will focus on further refining CPLODE with advanced adaptive mechanisms and exploring its application to a wider range of real-world optimization and feature selection tasks, including comparisons with traditional methods. We also intend to investigate the integration of machine learning and reinforcement learning techniques for more intelligent optimization strategies.

## Figures and Tables

**Table 1:** CEC2017 benchmark functions.

Function	Function Name	Class	Optimum
F1	Shifted and Rotated Bent Cigar Function	Unimodal	100
F2	Shifted and Rotated Zakharov Function	Unimodal	300
F3	Shifted and Rotated Rosenbrock’s Function	Multimodal	400
F4	Shifted and Rotated Rastrigin’s Function	Multimodal	500
F5	Shifted and Rotated Expanded Scaffer’s F6 Function	Multimodal	600
F6	Shifted and Rotated Lunacek Bi-Rastrigin Function	Multimodal	700
F7	Shifted and Rotated Non-Continuous Rastrigin’s Function	Multimodal	800
F8	Shifted and Rotated Lévy Function	Multimodal	900
F9	Shifted and Rotated Schwefel’s Function	Multimodal	1000
F10	Hybrid Function 1 (N = 3)	Hybrid	1100
F11	Hybrid Function 2 (N = 3)	Hybrid	1200
F12	Hybrid Function 3 (N = 3)	Hybrid	1300
F13	Hybrid Function 4 (N = 4)	Hybrid	1400
F14	Hybrid Function 5 (N = 4)	Hybrid	1500
F15	Hybrid Function 6 (N = 4)	Hybrid	1600
F16	Hybrid Function 6 (N = 5)	Hybrid	1700
F17	Hybrid Function 6 (N = 5)	Hybrid	1800
F18	Hybrid Function 6 (N = 5)	Hybrid	1900
F19	Hybrid Function 6 (N = 6)	Hybrid	2000
F20	Composition Function 1 (N = 3)	Composition	2100
F21	Composition Function 2 (N = 3)	Composition	2200
F22	Composition Function 3 (N = 4)	Composition	2300
F23	Composition Function 4 (N = 4)	Composition	2400
F24	Composition Function 5 (N = 5)	Composition	2500
F25	Composition Function 6 (N = 5)	Composition	2600
F26	Composition Function 7 (N = 6)	Composition	2700
F27	Composition Function 8 (N = 6)	Composition	2800
F28	Composition Function 9 (N = 3)	Composition	2900
F29	Composition Function 10 (N = 3)	Composition	3000

**Table 2:** Results of CPLODE and other algorithms on CEC2017.

	F1		F2		F3	
	Avg	Std	Avg	Std	Avg	Std
CPLODE	4.0920 × 10^3^	4.1750 × 10^3^	3.0002 × 10^2^	1.0051 × 10^−2^	4.5068 × 10^2^	3.4430 × 10^1^
PLO	1.1590 × 10^4^	2.4939 × 10^3^	2.7023 × 10^4^	5.6270 × 10^3^	4.7492 × 10^2^	1.1119 × 10^1^
SMA	2.5115 × 10^9^	9.8658 × 10^8^	3.7557 × 10^4^	8.5901 × 10^3^	6.1469 × 10^2^	5.3174 × 10^1^
WOA	2.6952 × 10^6^	1.5313 × 10^6^	1.4743 × 10^5^	6.0775 × 10^4^	5.6002 × 10^2^	3.6325 × 10^1^
GWO	2.1219 × 10^9^	1.3846 × 10^9^	3.4397 × 10^4^	1.0051 × 10^4^	7.0155 × 10^2^	4.0283 × 10^2^
MFO	1.2988 × 10^10^	6.9423 × 10^9^	7.7996 × 10^4^	6.2218 × 10^4^	1.1193 × 10^3^	6.5315 × 10^2^
SCA	1.2347 × 10^10^	2.1109 × 10^9^	3.5606 × 10^4^	5.4682 × 10^3^	1.3672 × 10^3^	2.2062 × 10^2^
FA	1.4659 × 10^10^	1.4882 × 10^9^	6.3838 × 10^4^	8.0840 × 10^3^	1.4042 × 10^3^	1.5042 × 10^2^
DE	2.3830 × 10^3^	4.8570 × 10^3^	2.1028 × 10^4^	5.1500 × 10^3^	4.9188 × 10^2^	1.0679 × 10^1^
	F4		F5		F6	
	Avg	Std	Avg	Std	Avg	Std
CPLODE	5.4831 × 10^2^	7.8331 × 10^0^	6.0000 × 10^2^	9.1994 × 10^−7^	7.8753 × 10^2^	8.8299 × 10^0^
PLO	5.5092 × 10^2^	7.4005 × 10^0^	6.0412 × 10^2^	6.4002 × 10^−1^	8.2325 × 10^2^	9.5400 × 10^0^
SMA	7.1420 × 10^2^	2.9243 × 10^1^	6.4455 × 10^2^	8.0890 × 10^0^	1.0707 × 10^3^	4.4764 × 10^1^
WOA	7.6420 × 10^2^	4.8394 × 10^1^	6.6890 × 10^2^	9.1396 × 10^0^	1.2308 × 10^3^	7.8906 × 10^1^
GWO	6.0427 × 10^2^	2.7433 × 10^1^	6.0927 × 10^2^	3.9375 × 10^0^	8.6044 × 10^2^	4.4426 × 10^1^
MFO	7.1517 × 10^2^	6.3055 × 10^1^	6.4193 × 10^2^	1.1270 × 10^1^	1.1255 × 10^3^	2.3548 × 10^2^
SCA	7.7692 × 10^2^	1.9626 × 10^1^	6.4836 × 10^2^	4.2782 × 10^0^	1.1213 × 10^3^	3.1481 × 10^1^
FA	7.6207 × 10^2^	9.3450 × 10^0^	6.4410 × 10^2^	2.6151 × 10^0^	1.3824 × 10^3^	3.1254 × 10^1^
DE	6.0933 × 10^2^	8.3500 × 10^0^	6.0000 × 10^2^	0.0000 × 10^0^	8.4291 × 10^2^	7.5915 × 10^0^
	F7		F8		F9	
	Avg	Std	Avg	Std	Avg	Std
CPLODE	8.4608 × 10^2^	8.3623 × 10^0^	9.0020 × 10^2^	2.6584 × 10^−1^	3.2070 × 10^3^	2.6162 × 10^2^
PLO	8.5204 × 10^2^	9.2515 × 10^0^	1.2746 × 10^3^	1.3036 × 10^2^	3.2575 × 10^3^	2.5941 × 10^2^
SMA	9.7055 × 10^2^	2.6769 × 10^1^	5.5826 × 10^3^	1.0568 × 10^3^	5.3672 × 10^3^	5.6679 × 10^2^
WOA	1.0136 × 10^3^	5.8658 × 10^1^	8.1838 × 10^3^	2.3689 × 10^3^	6.2527 × 10^3^	6.5665 × 10^2^
GWO	8.8678 × 10^2^	1.9861 × 10^1^	1.7494 × 10^3^	6.1671 × 10^2^	4.1153 × 10^3^	9.3870 × 10^2^
MFO	1.0139 × 10^3^	5.2970 × 10^1^	7.1350 × 10^3^	1.9884 × 10^3^	5.4931 × 10^3^	7.9595 × 10^2^
SCA	1.0478 × 10^3^	1.5537 × 10^1^	5.1287 × 10^3^	1.0492 × 10^3^	8.1431 × 10^3^	3.2193 × 10^2^
FA	1.0509 × 10^3^	1.4648 × 10^1^	5.4305 × 10^3^	5.1404 × 10^2^	7.9251 × 10^3^	3.8444 × 10^2^
DE	9.0849 × 10^2^	1.0072 × 10^1^	9.0000 × 10^2^	9.6743 × 10^−14^	5.9854 × 10^3^	1.9299 × 10^2^
	F10		F11		F12	
	Avg	Std	Avg	Std	Avg	Std
CPLODE	1.1229 × 10^3^	9.1014 × 10^0^	1.2004 × 10^5^	8.5831 × 10^4^	1.8444 × 10^4^	2.1474 × 10^4^
PLO	1.1581 × 10^3^	1.6584 × 10^1^	5.0177 × 10^5^	2.4604 × 10^5^	1.4198 × 10^4^	5.7243 × 10^3^
SMA	1.5322 × 10^3^	1.0156 × 10^2^	1.2983 × 10^8^	7.5032 × 10^7^	1.5058 × 10^6^	1.6082 × 10^6^
WOA	1.4913 × 10^3^	9.8025 × 10^1^	4.8594 × 10^7^	3.1101 × 10^7^	1.3602 × 10^5^	9.4765 × 10^4^
GWO	1.8226 × 10^3^	6.6599 × 10^2^	1.1275 × 10^8^	3.2956 × 10^8^	1.3704 × 10^7^	3.9247 × 10^7^
MFO	5.9355 × 10^3^	5.1442 × 10^3^	3.5650 × 10^8^	8.1328 × 10^8^	3.1216 × 10^8^	7.5404 × 10^8^
SCA	2.0945 × 10^3^	2.2808 × 10^2^	1.1051 × 10^9^	2.9299 × 10^8^	3.4755 × 10^8^	1.2259 × 10^8^
FA	3.2599 × 10^3^	5.7410 × 10^2^	1.4017 × 10^9^	3.0724 × 10^8^	6.1809 × 10^8^	1.7734 × 10^8^
DE	1.1611 × 10^3^	2.0851 × 10^1^	1.5862 × 10^6^	8.2773 × 10^5^	2.9151 × 10^4^	1.1875 × 10^4^
	F13		F14		F15	
	Avg	Std	Avg	Std	Avg	Std
CPLODE	1.4384 × 10^4^	1.0938 × 10^4^	1.2813 × 10^4^	1.3823 × 10^4^	1.9560 × 10^3^	1.3213 × 10^2^
PLO	7.9649 × 10^3^	4.7388 × 10^3^	4.5866 × 10^3^	1.2584 × 10^3^	1.9970 × 10^3^	1.1299 × 10^2^
SMA	1.8264 × 10^5^	9.5737 × 10^4^	2.0160 × 10^4^	9.2830 × 10^3^	2.8795 × 10^3^	3.3202 × 10^2^
WOA	5.4997 × 10^5^	7.1671 × 10^5^	6.1537 × 10^4^	5.5170 × 10^4^	3.5053 × 10^3^	4.9078 × 10^2^
GWO	1.0984 × 10^5^	1.8864 × 10^5^	3.5527 × 10^5^	8.3062 × 10^5^	2.3715 × 10^3^	2.8305 × 10^2^
MFO	2.7980 × 10^5^	6.5096 × 10^5^	5.6788 × 10^4^	4.2509 × 10^4^	3.0976 × 10^3^	3.6698 × 10^2^
SCA	1.1542 × 10^5^	6.3321 × 10^4^	1.1045 × 10^7^	8.4052 × 10^6^	3.6068 × 10^3^	2.2862 × 10^2^
FA	1.8689 × 10^5^	7.2559 × 10^4^	6.2655 × 10^7^	3.1177 × 10^7^	3.4083 × 10^3^	2.1965 × 10^2^
DE	6.4866 × 10^4^	5.4400 × 10^4^	7.6915 × 10^3^	4.1350 × 10^3^	2.0677 × 10^3^	1.3933 × 10^2^
	F16		F17		F18	
	Avg	Std	Avg	Std	Avg	Std
CPLODE	1.7990 × 10^3^	7.6248 × 10^1^	1.8955 × 10^5^	2.7639 × 10^5^	1.7549 × 10^4^	1.6480 × 10^4^
PLO	1.8186 × 10^3^	3.9754 × 10^1^	1.3635 × 10^5^	7.8731 × 10^4^	2.9860 × 10^3^	6.7273 × 10^2^
SMA	2.3156 × 10^3^	2.1345 × 10^2^	4.8558 × 10^5^	3.3512 × 10^5^	5.0410 × 10^5^	5.3037 × 10^5^
WOA	2.6334 × 10^3^	2.8446 × 10^2^	3.3275 × 10^6^	2.9399 × 10^6^	2.2655 × 10^6^	2.8705 × 10^6^
GWO	1.9486 × 10^3^	1.1480 × 10^2^	5.9764 × 10^5^	5.7548 × 10^5^	8.0009 × 10^5^	1.6674 × 10^6^
MFO	2.5460 × 10^3^	3.1139 × 10^2^	3.1751 × 10^6^	7.8848 × 10^6^	1.2902 × 10^7^	3.6770 × 10^7^
SCA	2.3934 × 10^3^	1.5338 × 10^2^	3.3788 × 10^6^	1.7657 × 10^6^	3.3238 × 10^7^	2.1205 × 10^7^
FA	2.4670 × 10^3^	1.2003 × 10^2^	4.3534 × 10^6^	1.9992 × 10^6^	9.2454 × 10^7^	3.1871 × 10^7^
DE	1.8421 × 10^3^	5.1530 × 10^1^	3.1519 × 10^5^	1.7642 × 10^5^	8.2470 × 10^3^	3.5513 × 10^3^
	F19		F20		F21	
	Avg	Std	Avg	Std	Avg	Std
CPLODE	2.1010 × 10^3^	6.2362 × 10^1^	2.3480 × 10^3^	9.5720 × 10^0^	3.4641 × 10^3^	1.3586 × 10^3^
PLO	2.1670 × 10^3^	5.1289 × 10^1^	2.3505 × 10^3^	7.3562 × 10^0^	2.3855 × 10^3^	4.1707 × 10^2^
SMA	2.4134 × 10^3^	1.2866 × 10^2^	2.4785 × 10^3^	2.4241 × 10^1^	3.2081 × 10^3^	1.4174 × 10^3^
WOA	2.6641 × 10^3^	2.0926 × 10^2^	2.5797 × 10^3^	6.7176 × 10^1^	6.2597 × 10^3^	2.2427 × 10^3^
GWO	2.4343 × 10^3^	1.2789 × 10^2^	2.3797 × 10^3^	2.6664 × 10^1^	4.5712 × 10^3^	1.3470 × 10^3^
MFO	2.6919 × 10^3^	2.6365 × 10^2^	2.5126 × 10^3^	4.5540 × 10^1^	6.5400 × 10^3^	9.2570 × 10^2^
SCA	2.6031 × 10^3^	1.4985 × 10^2^	2.5514 × 10^3^	2.2652 × 10^1^	7.6999 × 10^3^	2.5971 × 10^3^
FA	2.5986 × 10^3^	7.5334 × 10^1^	2.5382 × 10^3^	1.5345 × 10^1^	3.8226 × 10^3^	1.3006 × 10^2^
DE	2.1400 × 10^3^	7.0218 × 10^1^	2.4102 × 10^3^	9.0096 × 10^0^	3.7627 × 10^3^	1.6700 × 10^3^
	F22		F23		F24	
	Avg	Std	Avg	Std	Avg	Std
CPLODE	2.7010 × 10^3^	7.8806 × 10^0^	2.8693 × 10^3^	6.4910 × 10^0^	2.8878 × 10^3^	5.8163 × 10^−1^
PLO	2.6996 × 10^3^	8.5181 × 10^0^	2.8689 × 10^3^	7.2496 × 10^0^	2.8847 × 10^3^	1.2452 × 10^0^
SMA	2.8511 × 10^3^	3.1216 × 10^1^	3.0080 × 10^3^	3.0863 × 10^1^	3.0205 × 10^3^	5.3642 × 10^1^
WOA	3.0359 × 10^3^	8.6584 × 10^1^	3.1487 × 10^3^	8.0901 × 10^1^	2.9455 × 10^3^	3.1040 × 10^1^
GWO	2.7461 × 10^3^	3.6003 × 10^1^	2.9220 × 10^3^	4.7272 × 10^1^	2.9723 × 10^3^	2.5394 × 10^1^
MFO	2.8310 × 10^3^	3.4083 × 10^1^	2.9962 × 10^3^	3.4826 × 10^1^	3.3300 × 10^3^	4.3061 × 10^2^
SCA	2.9926 × 10^3^	2.3532 × 10^1^	3.1572 × 10^3^	3.5056 × 10^1^	3.1873 × 10^3^	5.5685 × 10^1^
FA	2.9135 × 10^3^	1.1764 × 10^1^	3.0649 × 10^3^	1.1714 × 10^1^	3.5444 × 10^3^	1.1607 × 10^2^
DE	2.7561 × 10^3^	8.9800 × 10^0^	2.9626 × 10^3^	1.2120 × 10^1^	2.8874 × 10^3^	3.4067 × 10^−1^
	F25		F26		F27	
	Avg	Std	Avg	Std	Avg	Std
CPLODE	4.1335 × 10^3^	9.3910 × 10^1^	3.2053 × 10^3^	1.0043 × 10^1^	3.1550 × 10^3^	6.6740 × 10^1^
PLO	3.9283 × 10^3^	4.7938 × 10^2^	3.2029 × 10^3^	4.0598 × 10^0^	3.2121 × 10^3^	9.0455 × 10^0^
SMA	5.2084 × 10^3^	5.6927 × 10^2^	3.2563 × 10^3^	2.0406 × 10^1^	3.4164 × 10^3^	4.4305 × 10^1^
WOA	7.7069 × 10^3^	9.8268 × 10^2^	3.3737 × 10^3^	1.0329 × 10^2^	3.3130 × 10^3^	3.1227 × 10^1^
GWO	4.5991 × 10^3^	3.2501 × 10^2^	3.2435 × 10^3^	2.1859 × 10^1^	3.4256 × 10^3^	7.9194 × 10^1^
MFO	6.0445 × 10^3^	6.8971 × 10^2^	3.2668 × 10^3^	3.0744 × 10^1^	4.4157 × 10^3^	9.8818 × 10^2^
SCA	6.9434 × 10^3^	2.7207 × 10^2^	3.4025 × 10^3^	3.8445 × 10^1^	3.8215 × 10^3^	1.3113 × 10^2^
FA	6.5170 × 10^3^	1.5945 × 10^2^	3.3344 × 10^3^	1.4804 × 10^1^	3.8905 × 10^3^	8.7135 × 10^1^
DE	4.6715 × 10^3^	6.3597 × 10^1^	3.2043 × 10^3^	3.6717 × 10^0^	3.1860 × 10^3^	4.7932 × 10^1^
	F28		F29			
	Avg	Std	Avg	Std		
CPLODE	3.4382 × 10^3^	9.2089 × 10^1^	1.0096 × 10^4^	3.3843 × 10^3^		
PLO	3.4618 × 10^3^	5.5133 × 10^1^	2.0740 × 10^4^	4.9557 × 10^3^		
SMA	4.0386 × 10^3^	2.0859 × 10^2^	5.2833 × 10^6^	2.8997 × 10^6^		
WOA	4.8405 × 10^3^	3.4810 × 10^2^	9.3134 × 10^6^	7.1522 × 10^6^		
GWO	3.7806 × 10^3^	1.6576 × 10^2^	4.5862 × 10^6^	3.5436 × 10^6^		
MFO	4.2868 × 10^3^	3.1200 × 10^2^	6.1200 × 10^5^	8.0878 × 10^5^		
SCA	4.6218 × 10^3^	2.1985 × 10^2^	7.0732 × 10^7^	2.6337 × 10^7^		
FA	4.7160 × 10^3^	1.2109 × 10^2^	9.4462 × 10^7^	3.0941 × 10^7^		
DE	3.5286 × 10^3^	7.2288 × 10^1^	1.2285 × 10^4^	3.3857 × 10^3^		
	Overall Rank					
	RANK	+/=−	AVG			
CPLODE	1	~	1.6552			
PLO	2	12/14/3	1.8621			
SMA	5	28/1/0	5.1379			
WOA	7	29/0/0	7.0000			
GWO	4	29/0/0	4.4483			
MFO	6	29/0/0	6.7586			
SCA	8	29/0/0	7.5517			
FA	9	29/0/0	7.7241			
DE	3	19/5/5	2.8621			

**Table 3:** The *p*-values of CPLODE versus other algorithms on CEC2017.

	**PLO**	**SMA**	**WOA**	**GWO**
F1	5.752 × 10^−6^	1.734 × 10^−6^	1.734 × 10^−6^	1.734 × 10^−6^
F2	1.734 × 10^−6^	1.734 × 10^−6^	1.734 × 10^−6^	1.734 × 10^−6^
F3	7.271 × 10^−3^	1.734 × 10^−6^	1.734 × 10^−6^	1.734 × 10^−6^
F4	1.414 × 10^−1^	1.734 × 10^−6^	1.734 × 10^−6^	1.734 × 10^−6^
F5	1.734 × 10^−6^	1.734 × 10^−6^	1.734 × 10^−6^	1.734 × 10^−6^
F6	1.734 × 10^−6^	1.734 × 10^−6^	1.734 × 10^−6^	2.127 × 10^−6^
F7	2.105 × 10^−3^	1.734 × 10^−6^	1.734 × 10^−6^	2.353 × 10^−6^
F8	1.734 × 10^−6^	1.734 × 10^−6^	1.734 × 10^−6^	1.734 × 10^−6^
F9	7.971 × 10^−1^	1.734 × 10^−6^	1.734 × 10^−6^	5.752 × 10^−6^
F10	1.921 × 10^−6^	1.734 × 10^−6^	1.734 × 10^−6^	1.734 × 10^−6^
F11	3.882 × 10^−6^	1.734 × 10^−6^	1.734 × 10^−6^	1.734 × 10^−6^
F12	7.655 × 10^−1^	1.734 × 10^−6^	1.921 × 10^−6^	1.921 × 10^−6^
F13	3.379 × 10^−3^	2.353 × 10^−6^	1.734 × 10^−6^	1.251 × 10^−4^
F14	5.710 × 10^−2^	4.492 × 10^−2^	1.734 × 10^−6^	1.238 × 10^−5^
F15	2.134 × 10^−1^	1.734 × 10^−6^	1.734 × 10^−6^	8.466 × 10^−6^
F16	8.972 × 10^−2^	1.734 × 10^−6^	1.734 × 10^−6^	1.799 × 10^−5^
F17	4.908 × 10^−1^	1.965 × 10^−3^	2.603 × 10^−6^	4.534 × 10^−4^
F18	2.843 × 10^−5^	3.182 × 10^−6^	1.734 × 10^−6^	4.729 × 10^−6^
F19	4.897 × 10^−4^	1.734 × 10^−6^	1.734 × 10^−6^	1.734 × 10^−6^
F20	1.470 × 10^−1^	1.734 × 10^−6^	1.734 × 10^−6^	3.182 × 10^−6^
F21	1.020 × 10^−1^	8.451 × 10^−1^	2.843 × 10^−5^	6.035 × 10^−3^
F22	8.290 × 10^−1^	1.734 × 10^−6^	1.734 × 10^−6^	6.984 × 10^−6^
F23	7.189 × 10^−1^	1.734 × 10^−6^	1.734 × 10^−6^	1.921 × 10^−6^
F24	2.127 × 10^−6^	1.734 × 10^−6^	1.734 × 10^−6^	1.734 × 10^−6^
F25	3.600 × 10^−1^	3.182 × 10^−6^	1.734 × 10^−6^	6.339 × 10^−6^
F26	4.779 × 10^−1^	1.734 × 10^−6^	1.734 × 10^−6^	2.127 × 10^−6^
F27	8.944 × 10^−4^	1.734 × 10^−6^	1.734 × 10^−6^	1.734 × 10^−6^
F28	7.521 × 10^−2^	1.734 × 10^−6^	1.734 × 10^−6^	1.921 × 10^−6^
F29	1.921 × 10^−6^	1.734 × 10^−6^	1.734 × 10^−6^	1.734 × 10^−6^
	MFO	SCA	FA	DE
F1	1.734 × 10^−6^	1.734 × 10^−6^	1.734 × 10^−6^	1.319 × 10^−2^
F2	1.734 × 10^−6^	1.734 × 10^−6^	1.734 × 10^−6^	1.734 × 10^−6^
F3	1.734 × 10^−6^	1.734 × 10^−6^	1.734 × 10^−6^	1.127 × 10^−5^
F4	1.734 × 10^−6^	1.734 × 10^−6^	1.734 × 10^−6^	1.734 × 10^−6^
F5	1.734 × 10^−6^	1.734 × 10^−6^	1.734 × 10^−6^	1.953 × 10^−3^
F6	1.734 × 10^−6^	1.734 × 10^−6^	1.734 × 10^−6^	1.734 × 10^−6^
F7	1.734 × 10^−6^	1.734 × 10^−6^	1.734 × 10^−6^	1.734 × 10^−6^
F8	1.734 × 10^−6^	1.734 × 10^−6^	1.734 × 10^−6^	6.104 × 10^−5^
F9	1.734 × 10^−6^	1.734 × 10^−6^	1.734 × 10^−6^	1.734 × 10^−6^
F10	1.734 × 10^−6^	1.734 × 10^−6^	1.734 × 10^−6^	6.339 × 10^−6^
F11	1.734 × 10^−6^	1.734 × 10^−6^	1.734 × 10^−6^	1.921 × 10^−6^
F12	1.734 × 10^−6^	1.734 × 10^−6^	1.734 × 10^−6^	2.183 × 10^−2^
F13	1.973 × 10^−5^	1.734 × 10^−6^	1.921 × 10^−6^	5.216 × 10^−6^
F14	5.216 × 10^−6^	1.734 × 10^−6^	1.734 × 10^−6^	4.653 × 10^−1^
F15	1.734 × 10^−6^	1.734 × 10^−6^	1.734 × 10^−6^	6.424 × 10^−3^
F16	1.734 × 10^−6^	1.734 × 10^−6^	1.734 × 10^−6^	6.836 × 10^−3^
F17	5.706 × 10^−4^	1.734 × 10^−6^	1.734 × 10^−6^	2.765 × 10^−3^
F18	1.127 × 10^−5^	1.734 × 10^−6^	1.734 × 10^−6^	1.657 × 10^−2^
F19	1.734 × 10^−6^	1.734 × 10^−6^	1.734 × 10^−6^	4.277 × 10^−2^
F20	1.734 × 10^−6^	1.734 × 10^−6^	1.734 × 10^−6^	1.734 × 10^−6^
F21	1.734 × 10^−6^	1.494 × 10^−5^	6.035 × 10^−3^	8.130 × 10^−1^
F22	1.734 × 10^−6^	1.734 × 10^−6^	1.734 × 10^−6^	1.734 × 10^−6^
F23	1.734 × 10^−6^	1.734 × 10^−6^	1.734 × 10^−6^	1.734 × 10^−6^
F24	2.127 × 10^−6^	1.734 × 10^−6^	1.734 × 10^−6^	3.162 × 10^−3^
F25	1.921 × 10^−6^	1.734 × 10^−6^	1.734 × 10^−6^	1.734 × 10^−6^
F26	1.734 × 10^−6^	1.734 × 10^−6^	1.734 × 10^−6^	8.130 × 10^−1^
F27	1.734 × 10^−6^	1.734 × 10^−6^	1.734 × 10^−6^	6.511 × 10^−2^
F28	1.734 × 10^−6^	1.734 × 10^−6^	1.734 × 10^−6^	8.944 × 10^−4^
F29	1.734 × 10^−6^	1.734 × 10^−6^	1.734 × 10^−6^	5.193 × 10^−2^

**Table 4:** Detailed description of datasets.

Datasets	Samples	Features	Classes
Breast cancer	286	9	2
Heart EW	270	13	2
Lymphography	148	18	4
Hepatitis_full_data	155	19	2
Glass	214	9	6
Heart	303	13	5
Thyroid_2class	187	8	2
Leukemia	72	7130	2
Vote	534	16	2
Segment	2310	18	7

**Table 5:** Classification error rates of different algorithms.

Function	CPLODE	BPSO	BGSA	BALO	BBA	BSSA
Breast cancer	9.21 × 10^−2^	9.76 × 10^−2^	9.91 × 10^−2^	9.36 × 10^−2^	1.62 × 10^−1^	9.42 × 10^−2^
	7.68 × 10^−3^	9.52 × 10^−3^	1.08 × 10^−2^	1.29 × 10^−2^	1.38 × 10^−2^	1.36 × 10^−2^
Heart EW	7.68 × 10^−2^	8.10 × 10^−2^	8.75 × 10^−2^	8.36 × 10^−2^	1.72 × 10^−1^	8.32 × 10^−2^
	2.32 × 10^−2^	4.21 × 10^−2^	3.67 × 10^−3^	3.52 × 10^−2^	2.58 × 10^−2^	3.14 × 10^−3^
Lymphography	1.82 × 10^−2^	2.42 × 10^−2^	2.38 × 10^−2^	1.97 × 10^−2^	6.52 × 10^−2^	2.01 × 10^−2^
	5.12 × 10^−3^	(4.23 × 10^−3^)	(3.25 × 10^−3^)	(2.46 × 10^−3^)	(1.23 × 10^−3^)	(4.02 × 10^−3^)
Hepatitis_full_data	1.28 × 10^−2^	1.93 × 10^−2^	2.57 × 10^−2^	9.42 × 10^−3^	2.37 × 10^−1^	1.82 × 10^−2^
	(1.47 × 10^−3^)	(3.23 × 10^−2^)	(5.36 × 10^−3^)	(1.86 × 10^−3^)	(8.45 × 10^−2^)	(4.89 × 10^−3^)
Glass	9.86 × 10^−2^	1.17 × 10^−1^	1.07 × 10^−1^	1.21 × 10^−1^	2.92 × 10^−1^	1.06 × 10^−1^
	(6.49 × 10^−2^)	(4.25 × 10^−2^)	(4.99 × 10^−2^)	(5.44 × 10^−2^)	(1.08 × 10^−1^)	(4.93 × 10^−2^)
Heart	6.18 × 10^−2^	7.03 × 10^−2^	5.58 × 10^−2^	6.97 × 10^−2^	2.63 × 10^−1^	6.28 × 10^−2^
	(3.54 × 10^−2^)	(4.43 × 10^−2^)	(5.21 × 10^−2^)	(3.39 × 10^−2^)	8.96 × 10^−2^)	(3.49 × 10^−2^)
Thyroid_2class	1.89 × 10^−1^	2.01 × 10^−1^	2.14 × 10^−1^	2.08 × 10^−1^	3.21 × 10^−1^	2.17 × 10^−1^
	5.36 × 10^−2^	(6.49 × 10^−2^)	(6.85 × 10^−2^)	(7.31 × 10^−2^)	(8.81 × 10^−2^)	(7.59 × 10^−2^)
Leukemia	0.00 × 10^0^	0.00 × 10^0^	1.05 × 10^−1^	0.00 × 10^0^	1.65 × 10^−2^	0.00 × 10^0^
	(0.00 × 10^0^)	(0.00 × 10^0^)	(4.21 × 10^−2^)	(0.00 × 10^0^)	(4.91 × 10^−2^)	(0.00 × 10^0^)
Vote	2.13 × 10^−2^	2.76 × 10^−2^	2.437 × 10^−2^	8.96 × 10^−2^	3.72 × 10^−2^	3.92 × 10^−2^
	1.62 × 10^−2^	3.68 × 10^−3^	9.86 × 10^−2^	2.83 × 10^−2^	3.13 × 10^−2^	4.07 × 10^−3^
Segment	2.26 × 10^−2^	2.35 × 10^−2^	2.48 × 10^−2^	2.42 × 10^−2^	4.23 × 10^−2^	2.88 × 10^−2^
	3.65 × 10^−3^	6.58 × 10^−3^	7.38 × 10^−3^	6.74 × 10^−3^	9.46 × 10^−3^	5.33 × 10^−3^

**Table 6:** Number of selected features by different algorithms.

Function	CPLODE	BPSO	BGSA	BALO	BBA	BSSA
Breast cancer	4.2	4.8	5.8	4.8	5.3	5.8
	(1.35)	(1.47)	(1.08)	(0.66)	(0.93)	(1.36)
Heart EW	5.3	5.5	6.2	5.3	4.4	5.7
	(0.85)	(1.44)	(0.96)	(0.84)	(1.21)	(0.93)
Lymphography	4.2	4.4	4.3	4.8	8.4	4.8
	(0.99)	(0.68)	(1.17)	(1.23)	(2.67)	(2.05)
Hepatitis_full_data	5.6	6.3	4.2	6.3	6.1	5.3
	(2.32)	(1.25)	(1.71)	(2.21)	(2.37)	(2.56)
Glass	3.7	3.9	4.6	3.9	4.1	4.3
	(0.92)	(0.74)	(1.36)	(0.67)	(1.53)	(0.87)
Heart	6.4	6.1	6.4	5.9	5.7	6.2
	(0.63)	(0.78)	(0.62)	(1.46)	(1.05)	(1.17)
Thyroid_2class	4.1	5.5	4.3	4.0	3.9	4.2
	(0.73)	(1.28)	(0.76)	(1.20)	(1.34)	(1.37)
Leukemia	1879.3	5236.4	2531.7	2657.0	3146.3	2768.1
	(26.32)	(67.45)	(23.32)	(46.44)	(38.52)	(94.68)
Vote	3.8	3.2	3.1	2.7	6.3	4.4
	(1.62)	(1.33)	(1.67)	(1.24)	(1.35)	(2.50)
Segment	5.2	6.3	5.4	5.3	7.6	6.2
	(0.83)	(0.94)	(0.99)	(1.48)	(1.76)	(1.66)

## Data Availability

The raw data supporting the conclusions of this article will be made available by the authors on request.
